# Validation of Motor Outcome Measures in Myotonic Dystrophy Type 2

**DOI:** 10.3389/fneur.2020.00306

**Published:** 2020-04-21

**Authors:** Federica Montagnese, Emanuele Rastelli, Nina Khizanishvili, Roberto Massa, Kristina Stahl, Benedikt Schoser

**Affiliations:** ^1^Department of Neurology, Friedrich-Baur-Institute, Klinikum der Universität, Ludwig-Maximilians-University, Munich, Germany; ^2^Department of Neurology, City Hospital Soest, Soest, Germany; ^3^Neuromuscular Diseases Unit, Department of Systems Medicine, Tor Vergata University of Rome, Rome, Italy

**Keywords:** myotonic dystrophy type 2, PROMM, DM2, outcome measures, patient reported outcomes, validation

## Abstract

**Introduction:** Myotonic dystrophy type 2 (DM2) lacks disease-specific, validated, motor outcome measures (OMs), and patients' reported outcomes (PROs). This represents a limit for the monitoring of disease progression and treatment response. Our aim was to identify the most appropriate OMs to be translated in clinical practice and clinical trials on DM2. This study has been registered on clinicaltrials.gov NCT03603171 (https://clinicaltrials.gov/ct2/show/NCT03603171).

**Methods:** Sixty-six patients with genetically confirmed DM2 underwent a baseline and a follow-up visit after 1 year. The tested OMs included: hand opening time, pressure pain threshold (PPT), manual muscle testing (MMT), hand held dynamometry (HHD), scale for the assessment and rating of ataxia (SARA), quantitative motor function test (QMFT), gait stairs Gowers chair (GSGC), 30-s sit to stand test, functional index 2 (FI-2) and 6MWT. The PROs included DM1-Active-C, Rasch-built Pompe-specific activity scale (R-Pact), fatigue and daytime sleepiness (FDSS), brief pain inventory short form (BPI-sf), myotonia behavior scale (MBS), and the McGill pain questionnaire.

**Results:** All patients completed the MBS and the results correlated well with the hand-opening time. The PPT showed a low reliability, no correlation with pain questionnaires, and did not differentiate patients with or without myalgia. Both muscle strength assessments, MMT and HHD, showed good construct validity. The QMFT showed an acceptable ceiling effect (14.5%), good convergent and differential validity and performed overall better than GSGC. The SARA score showed high flooring effect and is not useful in DM2. 6MWT proved a valid outcome measure in DM2. The 30-s sit to stand is a feasible test with good convergent validity, showing a flooring effect of 20% as it cannot be used in more severely affected patients. The FI-2 is time-consuming and has a high ceiling effect. At the 1-year visit the only assessments able to detect a worsening of DM2 were HHD, QMFT, and 6MWT, which are the most sensitive to change, and therefore clinically meaningful OMs in DM2.

**Conclusion:** The clinical meaningful motor outcome measures that best depict the multifaceted phenotype of DM2 and its slow progression are MBS, MMT, or HHD (depending on the clinical setting), QMFT, and the 6MWT.

## Introduction

Myotonic dystrophies types 1 (DM1) and 2 (DM2) are autosomal dominant, multisystemic diseases, due to CTG- or CCTG-repeat expansion mutations in *DMPK* and *CNBP*, respectively (1, 2). DM2 has, compared to DM1, a lower prevalence worldwide and a milder phenotype spectrum with a slower progression. The first symptoms usually occur after the 3rd to 4th decade of life, when patients complain of myalgia, mostly exercise-induced, and proximal as well as axial weakness; a smaller proportion of patients also show signs and symptoms of a usually mild myotonia. As in DM1, DM2 patients also present a multisystemic involvement with higher risk for developing heart diseases, cataract and endocrine dysfunction in comparison to the general population (3, 4). The clinical progression is very slow and only a minority of patients will require walking aids during the course of the disease (3). Causal therapies are not yet available; however, the first clinical trials are on their way for DM1 and expectantly for DM2. In order to evaluate the efficacy of any future therapies, reliable and disease-specific outcome measures are necessary to detect any clinically relevant changes of patients under treatment. In this regard, several functioning and disability scales have already been tested and validated in DM1 patients (6-min walk test, muscle impairment rating scale, DM1-Active-C, scale for assessment and rating of ataxia) (5–8). Furthermore, four workshops with international experts on myotonic dystrophies, focusing on the selection of outcome measure in DM1, took place between November 2011 and June 2019 (OMMYD-1, -2, -3, -4) (9–11). During these meetings, experts identified those motor outcome measures that should be adopted by every laboratory to standardize the clinical assessment of DM1 patients: 6-min walking test (6MWT), 10-meter walk test (10MWT), Nine Holes Peg Test (NHPT), the 30-s sit to stand test, manual muscle testing (MMT), and quantitative muscle testing (QMT) (9–11). Comparable recommendations are not available for DM2 and some of these tests are not suitable in DM2 patients due to their milder and different phenotype. A recent systematic review highlighted the lack of valid and reliable outcome measures to be adopted in DM2 patients and invited to fill this gap (12). The aim of our study is therefore to test how the most widely used motor outcome measures would perform in the DM2 population, in order to identify a feasible test battery to be adopted in clinical trials and patients' follow-up.

## Materials and Methods

This is a monocentric, longitudinal, prospective, observational study registered on clinicaltrials.gov as “Clinical Outcome Measures in Myotonic Dystrophy Type 2,” identification number NCT03603171. The study has been approved by the local ethic committee (Project 18-266) and was conducted in accordance with the Helsinki declaration; all participants gave written informed consent before study participation.

### Patients

Patients were informed about the study using the German-Swiss registry for Myotonic Dystrophies (DM-Register) (https://www.dm-registry.org/de/). The following inclusions' criteria were considered: (a) genetically confirmed diagnosis of DM2; (b) patient able to provide informed consent; (c) age between 18 and 80 years; (d) German-speaking patients. The exclusion criteria were: (i) patient unable to provide the informed consent; (ii) presence of invalidating co-morbidities possibly influencing motor outcomes (e.g., previous stroke, orthopedic conditions, heart failure). A baseline (V0) and a follow-up visit at 1 year (V1) were performed. At V0 patients fulfilled, with the help of the investigators, a general questionnaire on DM2 encompassing the following data: demographics, family history, age at onset, symptoms at onset, age at diagnosis, current muscular complaints, comorbidities, current medications, use of walking aids. Thereafter, a standard neurological examination (including MMT grading 0–5 and search for action/percussion myotonia) and the test battery were performed. At V1 only the test battery was repeated.

### Test Battery

The test battery included a set of motor outcome measures (OMs) and patients' reported outcomes (PROs). The OMs and PROs were selected considering the OMMYD recommendations for DM1 but also including some additional OMs adopted in myopathies, that share some similar clinical features with DM2 (e.g., Pompe disease).

The OMs included: hand opening time, pressure pain threshold (PPT), manual muscle testing (MMT, grading 0–10), hand held dynamometry (HHD), scale for the assessment and rating of ataxia (SARA), quantitative motor function test (QMFT), gait stairs Gowers chair (GSGC), 30-s sit to stand test, functional index version 2 for upper limbs (FI-2) and 6MWT.

The PROs included DM1-Active-C, Rasch-built Pompe-specific activity scale (R-Pact), fatigue and daytime sleepiness scale (FDSS), brief pain inventory short form (BPI-sf), myotonia behavior scale (MBS), INQoL version 2.0 “muscle locking” sub-part, McGill pain questionnaire.

The assessments were performed in the following order: (1) Filling out the PROs, (2) MBS and hand opening time, (3) PPT, (4) MMT and HHD, (5) SARA scale, (6) QMFT, (7) GSGC, (8) 30 s sit to stand, (9) FI-2, (10) 6MWT. At the beginning, the tests were demonstrated and explained, in order to allow patients to become familiar with the different tasks. Patients were instructed to give the maximum effort; they were however allowed to interrupt a test or the whole assessment if needed (excessive fatigue, unbearable myalgia). Adequate resting intervals were observed between motor tests. The same evaluator performed all OMs at V0 (E.R.) other evaluators performed the OMs at V1 (F.M., N.K.). All evaluators were trained for the proper and standardized conduct of the above-mentioned OMs. All assessments were performed in about 3 h.

The hand opening time is a simple test to assess grip myotonia; participants were asked to make a tight fist for 5 s and then rapidly open the hand. Five trials were performed and the time was measured with a stopwatch. The average value was calculated (13). The PPT was assessed by a manual pressure algometer (Medictronics 22–42Algo): the rubber tip of the algometer was placed on standardized muscles (tibialis anterior, vastus lateralis, deltoideus and finger extensor), than the pressure was increased at approximately 100 g/s continuously until the subject perceived pain and said “stop.” Two measurements for each location were performed; a third measurement was obtained if there was a difference >10%. The average value was retained for statistical analysis. For manual muscle testing (MMT) we have used the MRC modified version with grades from 0 to 10 and examined the following muscle groups: neck flexors, neck extensors, shoulder abductors, elbow flexors and extensors, wrist flexors and extensors, hip flexors and extensors, knee flexors and extensors, ankle dorsiflexors, and plantar flexors. Handheld dynamometry (HHD) was performed with microFET-2 assessing the same muscles as above. Two measurements for each side with 20–30 s rest between trials were performed. A third measurement was performed in case of difference > 10%. The average value was retained for statistical analysis. SARA is an 8-item scale, yielding a total score ranging from 0 (no ataxia) to 40 (most severe ataxia), this has been recently proved to be valid in DM1 (6). The QMFT is a 16-items test validated to assess motor function in Pompe disease (14). The GSGC Scale is used in Pompe disease providing a quantitative (timed performance) and qualitative evaluation (severity grades) of 4 motor performances usually compromised in patients with proximal muscle weakness: G = Gait by walking for 10 m, S = climbing 4 steps, G = Gowers' maneuver, C = rising from a Chair (15). The 30-s sit to stand test (30CST) assesses lower extremity motor function and endurance. It is performed on a 43 cm high chair without armrest; the participant is seated with the arms crossed and held against the chest and has to perform as many full stands as possible within 30 s (16). The FI-2 has been developed for myositis patients, in the present study only the upper extremities evaluation was used. It consists of two parts: part (1) patients are requested to sit without back support and to perform as many full shoulder abduction movements (each arm separately) as possible without using compensatory muscles and with a pace of 40 beats/min; part (2) patient are seated with back support and have to perform shoulder flexion movements with 1 kg weight cuff around the wrist, keeping the same pace of 40 beats/min (17). The test will be stopped for one of the following reasons: (i) the patient was able to correctly perform the maximum number of 60 repetitions, (ii) the patient could not maintain the pace of 40 beats/min due to fatigue/weakness, (iii) compensatory muscles were used due to fatigue/weakness, (iv) the patient decides to stop the test due to excessive fatigue or pain. The 6MWT was performed following the American Thoracic Society (ATS) guidelines (18). A pre-test resting period of about 15 min was observed. Patients walked up and down a straight corridor along a 25-meter line as fast as possible, but without running, for 6 min. Patients could make a pause if needed, the distance walked at the end of the test was recorded. The Enright formula was used to calculate the percent of the predicted distance from normative values (19).

### Statistical Analysis

The data were analyzed with IBM SPSS statistics software v25.0. Data normality was assessed with the Shapiro–Wilk test. Our data were summarized either as mean (±SD) or median (inter quartile range, IQR), according to the distribution of each continuous variable. Accordingly, the *t*-Student test and the Mann-Whitney *U* test were used, as appropriate, to compare differences among two groups. Fisher's exact tests were used to compare categorical variables across patient subgroups. As no gold standard exist to evaluate DM2 patients, criterion validity was assessed by testing whether a given test would correlate with another test assessing a similar parameter. Correlations studies were performed with the Pearson's correlation test for continuous variables and Spearman's correlation coefficient for ranked variables. Construct validity was accepted if values fell between 0.4 and 0.9. Ceiling and flooring effects were calculated for each test. To test differences among multiple groups the one-way analysis of variance has been used followed by *post-hoc* Bonferroni correction. To compare the results of V0 and V1 the t-student for paired cohorts and the Wilcoxon signed-rank test have been used for parametric and non-parametric variables, respectively. Internal consistency was deemed acceptable with a Cronbach' alpha coefficient of 0.7 or higher. All statistical tests were performed two-sided and a *p* < 0.05 was considered significant.

## Results

The total number of genetically confirmed DM2 patients recruited in this study was 71. Of them, 66 patients fulfilled the inclusion/exclusion criteria. Demographics are reported in Table 1. A subgroup of n=49 patients underwent a follow-up visit after 10.08 ± 1.8 months. All patients completed the test battery. 17% of patients complained of muscle pain for a couple of days following the study visits. This, together with the absence of funding for the study, accounted for the relatively high drop-out of patients at V1 (25%).

**Table 1:** Demographics and baseline characteristics of DM2 patients.

	**Total**
Patients number *n* (%)	66; females 44/66 (62.9%)
Age (mean ± SD)	54.8 ± 12.4 (females 54.4 ± 12.8; males 54.6 ± 12.4; p = 0.763)
BMI (mean ± SD)	27.2 ± 6.2
Age at onset (mean ± SD)	35.6 ± 13.7
Disease duration (mean ± SD)	19.1 ± 12.1
**MUSCULAR SYMPTOMS AT V0**	
Weakness (%)	43 (65.1)
Myalgia (%)	37 (56)
Myotonia (%)	13 (19.6)
**NEED OF WALKING AID**	
No (%)	45 (68.1)
Yes (%)	21 (31.8)
Wheelchair	5
Walker	5
Cane	7
Other (e.g., Foot extensor orthesis)	4

### Assessment of Myotonia

The results of the MBS were: 22/66 grade 0 (no stiffness), 13/66 grade 1, 15/66 grade 2, 16/66 grade 3, 3/66 grade 4 and nobody answered with grade 5 (incapacitating stiffness). Overall 75.7 % of patients referred either no stiffness or a very mild episodic stiffness not impairing daily activities. The mean value of MBS at V0 was 1.51 ± 1.29; the results of V1 did not significantly differ from those at V0 being the mean MBS 1.52 ± 1.18 (*p* = 0.584). As regards the hand opening time the mean value was 0.62 s (range 0.12–3.4 s): 29/66 (44%) patients had an hand opening time <0.3 s (upper limit of normal). There was a positive correlation between MBS and hand opening time (0.490, *p* < 0.01). No significant difference was observed for MBS and hand opening time between V0 and V1 (Table 2). The only correlation we could find as regards MBS was a positive correlation with pain assessments (McGill 0.406 and BPI-sf 0.431) but not PPT. The INQoL V2.0 was answered by 59/66 patients, the remaining 7 patients argued that “muscle locking” did not reflect their complaints. The median score of the three questions was 7 (IQR 4-12) at V0 and no significant change was observed at V1. This questionnaire showed no significant correlation with MBS and pain assessments.

**Table 2:** Comparison of results between V0 and V1.

**Outcome measure**	***n***	**Flooring effect %**	**Ceiling effect %**	**Baseline (V0)**	**Follow-up (V1)**	***p*-value (V0-V1)**
MMT Sumscore median (IQR)	66	-	-	116.6 (109.6–119.2)	115.5 (110.2–118.7)	0.39
HHD Sumscore median (IQR)	66	-	-	318.4 (265–380,7)	312.9 (232.6–359.5)	0.000^*^
Hand opening time median (range)	66	-	-	0.6 (range 0.12–3.4)	0.45 (range 0.1–2.12)	0.33
MBS median (IQR)	66	33.3	0	0 (0–3)	0 (0–3)	0.48
SARA median (IQR)	66	50.7	0	0 (0–3) 1.9 ± 2.8	0 (0–3) 1.5 ± 2.37	0.15
QMFT median (IQR)	66	0	14.5	53.5 (40–60.5)	47 (35.5–59)	0.001^*^
GSGC median (IQR)	66	36.3	0	6.5 (4–13)	7.5 (5–12.2)	0.13
30-s sit to stand median (IQR)	53	19.6	-	9 (3.5–14.511)	8 (4-14)	0.07
FI-2 median (IQR)	64	-	32.8	60 (28-60)	60 (26-60)	0.13
6MWT mean ± SD	60	-	-	459.05 ± 153.6	424.89 ± 174.4	0.003^*^

*Bold values indicate the statistically significant*.

* *p < 0.05*.

### Assessment of Myalgia

**Figure 1:**
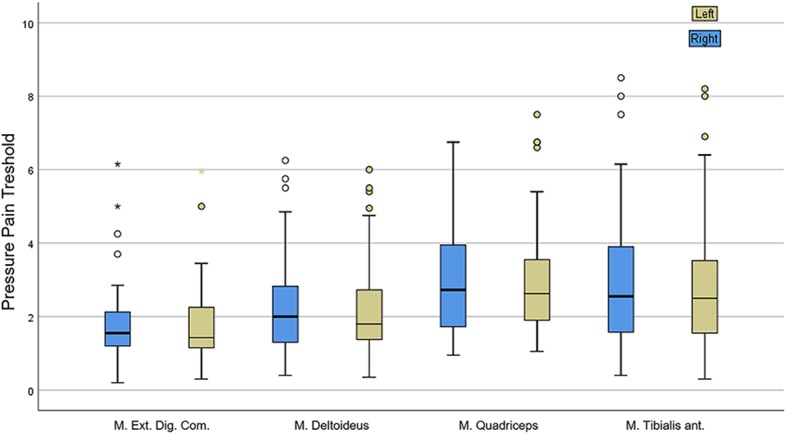
Results of pressure pain threshold (PPT) assessment showing great variability within single muscles.

We adopted the definition of Fischer et al. for hyperalgesia, in which a PPT below 3 kg/cm^2^ or a difference from the contralateral PPT exceeding 2 kg/cm^2^ indicates mechanical hyperalgesia (20). We found a total of 73.8% patients suffering from hyperalgesia. No difference between muscles of both body sides were observed. PPT did not correlate with any of the pain questionnaires, and comparing the scores of patients with or without myalgia no significant difference was found. There was a high variability between repeated measurements in the same patient and within examined muscles (Figure 1).

### Assessment of Muscle Strength

The results of each examined muscle with MMT and HHD are reported in Table 3. The weakest muscles were, as expected, the neck flexors and hip flexors. We found a strong correlation between the two assessment methods (0.60–0.79) especially for neck flexors, hip flexors and extensors. For all other muscle groups there still was a moderate correlation (0.40–0.59). Also, a high correlation was found between same muscles examined at both sides thus confirming that the muscle weakness in DM2 is largely symmetrical. Comparing the results of MMT at V0 and V1 no significant difference was observed (0.399). A statistically significant reduction of the total HHD score was however observed comparing the results of V0 and V1 (*p* < 0.000) (Table 2). Strong correlations were found between MMT and HHD with motor function measures QMFT, 6MWT, GSGC, 30 s sit to stand.

**Table 3:** Muscle strength assessed by manual muscle testing (MMT) and hand-held dynamometry (HHD) at baseline (V0).

	**MMT-10**				**HHD**				**Correlations MMT-HHD**
	**Min**	**Max**	**Mean**	**± SD**	**Min**	**Max**	**Mean**	**±SD**	** = strong * = moderate
NeckFlex	**2.0**	**5.0**	**4.4**	**0.6**	3.1	17.7	9.3	3.7	0.637**
NeckExt	4.0	5.0	4.9	0.4	4.0	21.2	12.8	4.1	0.521*
ShoulderAbd R	3.2	5.0	4.7	0.4	3.5	20.5	9.7	3.5	0.583*
ShoulderAbd L	3.2	5.0	4.6	0.4	4.3	18.9	9.5	3.5	0.646**
Elb_flex_R	4.2	5.0	4.9	0.2	7.6	28.7	15.0	4.0	0.597*
Elb_flex_L	4.0	5.0	4.9	0.2	7.8	25.6	14.1	4.4	0.550*
Elb_ext_R	4.0	5.0	4.7	0.4	4.1	19.2	9.7	3.8	0.542*
Elb_ext_L	4.0	5.0	4.7	0.4	4.0	20.1	9.6	3.7	0.615**
Wri_ext_R	4.2	5.0	5.0	0.2	3.4	24.9	12.5	4.0	0.490*
Wri_ext_L	4.2	5.0	4.9	0.2	3.0	24.8	12.1	4.4	0.481*
Wri_flex_R	4.2	5.0	5.0	0.1	5.7	24.0	13.0	4.2	0.373
Wri_flex_L	3.8	5.0	4.9	0.2	0.0	24.6	12.8	5.0	0.462*
HipFlex_R	**3.0**	**5.0**	**4.5**	**0.6**	1.8	29.8	15.6	6.5	0.804**
HipFlex_L	**3.0**	**5.0**	**4.5**	**0.6**	3.0	33.3	15.7	6.2	0.774**
HipExt_R	2.2	5.0	4.7	0.6	4.2	31.2	14.2	6.0	0.779**
HipExt_L	2.2	5.0	4.7	0.6	4.3	29.6	14.2	5.7	0.718**
KneeFlex_R	3.0	5.0	4.8	0.4	4.6	32.2	15.2	5.6	0.508*
KneeFlex_L	3.0	5.0	4.8	0.4	4.4	30.4	15.0	5.6	0.526*
KneeExt_R	3.8	5.0	4.8	0.4	7.3	46.8	19.7	7.9	0.588*
KneeExt_L	3.8	5.0	4.8	0.4	5.8	52.6	19.7	8.2	0.595*
AnkleDor_R	3.0	5.0	4.9	0.3	1.1	25.1	14.3	5.5	0.427*
AnkleDor_L	3.0	5.0	4.9	0.3	1.3	26.8	14.4	5.8	0.473*
AnklePla_R	4.0	5.0	5.0	0.1	4.7	42.5	23.4	5.7	0.242
AnklePla_L	4.0	5.0	5.0	0.2	5.9	41.0	23.2	5.5	0.339

*Bold values indicate the weakest muscles*.

### Balance Assessment

The mean SARA sum score for this study at V1 was 1.9±2.8 (median 0 IQR 0-3). 50.7% of the patients scored 0 at the SARA sum score. Moderate correlations were observed with QMFT, GSGC, and 6MWT. Chronbach's alpha was good 0.727. After principal component analysis the first 3 items had an eigenvalue higher than 1, in particular the first item “gait” had an eigenvalue of 3.17 and was responsible for 39% of the variance. By eliminating items 4, 5, and 6 (speech disturbances, finger chase and finger-nose, respectively), there would be a slight increase in Chronbach's up to 7.39. No relevant change was observed between V0 and V1 (Table 2).

### Motor Function Assessments

**Figure 2:**
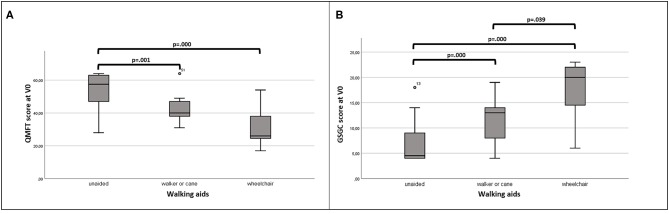
Discrimination validity of QMFT **(A)** and GSGC **(B)**.

All patients could complete the QMFT test. We found a ceiling effect of 14.5%. The median value at V0 was 53.5 (40–60.5), at V1 47 (35.5–59) (*p* = 0.001) showing a statistically significant worsening at V1. The QMFT showed an excellent convergent validity with strong correlations with DM1-Active-C, R-PACT, GSGC, 6MWT, 30 s sit to stand, and moderate correlation with FI-2. QMFT also significantly correlated with disease duration and age. Chronbach's alpha was 0.951. QMFT was able to differentiate DM2 patients not needing a walking aid from patients using a cane/walker or a wheelchair (Figure 2). As regards the GSGC, the median score was 6.5 (4–13) at V0 and 7.5 (5–12.5) at V1. This scale performed similarly to QMFT as regards convergent validity (significant correlations with age, DM1-Active-C and R-PACT, QMFT, 6MWT, 30 s sit to stand, and FI-2) and differential validity (Figure 2B). However, there was a very high flooring effect of 36%. No statistically significant difference was observed at V1 for GSGC.

### Muscle Endurance

The 6MWT was performed by 60/66 of DM2 patients (90.9%); the results were compared to normative data for adults adopting the Enright formula and expressed as percentage of predicted value. 13/60 (21%) patients performed better than predicted values for age and gender (range 101.9–114.9%), whereas the remaining 47 patients walked a mean distance of 69% ± 24 of predicted (range 18–99%). Convergent validity of 6MWT with several other outcome measures is shown in Table 4 (strong positive correlation with DM1-Active-C, R-PACT, QMFT, and FI-2; strong negative correlation with GSGC), a moderate positive correlation was found with manual muscle strength at lower limbs in particular hip flexors and knee extensors. No significant correlation was noticed with MBS. No significant difference was found between patients with and without myalgia (*p* = 0.560). A statistically significant decrease of 34,16 m in the distance walked on 6MWT has been found at V1 (Table 2).

**Table 4:** 6MWT convergent validity: correlations with other motor function outcome measures and patients reported outcomes.

**6MWT**	***n***	**Pearson/Spearmann correlation coefficient**	***p*-value**
DM-1 Active-C	60	0.798	0.000^*^
R-Pact	60	0.779	0.000^*^
QMFT	60	0.868	0.000^*^
FI-2	60	0.676	0.000^*^
GSGC	60	−0.858	0.000^*^
30sec sit to stand	60	0.733	0.000^*^
MMT LL sum score	60	0.492	0.000^*^
FDSS	60	−0.362	0.003^§^
BPI - SF	60	−0.453	0.001^§^
MBS	60	−0.218	0.084

* p < 0.001,

§ *p < 0.05. QMFT, quantitative motor function test; FI-2, functional index 2; GSGC, Gait Stairs Gowers Chair; MMT LL, Manual muscle test sum score of lower limbs; FDSS, fatigue and daytime sleepiness score; BPI-SF, Brief Pain Inventory Short Form; MBS, Myotonia Behavior Scale*.

The 30 s sit to stand test could be performed by 53/66 patients, which means that a flooring effect (score=0) was observed in 13/66 patients (19.6%). No significant difference was seen at V1 (Table 2). Strong to moderate correlations were observed with age, DM1-Active, R-Pact, QMFT, GSGC, and 6MWT.

The FI-2 test for upper limbs was performed in 64/66 patients, the remaining 2 patients could only be examined on the left side due to previous luxation or operation of the right shoulder. 21/64 patients completed the test on both sides corresponding to a ceiling effect of 32.8%. The remaining 77.2% of patients performed a mean of 37.17 ± 19.06 repetitions on the right arm and 35.8 ± 18.32 on the left (*p* = 0.08), whereas in the second part of the test they scored a mean of 23.62 ± 16.24 right and 21.42 ± 15.74 left (*p* = 0.06). This test correlated with DM1 Active-C, R-Pact, BPI-sf, QMFT, GSGC and with MMT of shoulder abduction. No significant difference was seen between patients with and without myalgia. At V1, no statistically significant change was observed.

Tests of endurance, in particular 6MWT, are influenced by the presence of abnormal pulmonary function or respiratory insufficiency. During disease history collection, patients were asked for symptoms of respiratory involvement or use of non-invasive ventilation (NIV). Five patients of the entire cohort reported of exercise-induced dyspnoe and 3 patients have OSAS treated with NIV (8/66, 12%). Out of the 60 patients that underwent 6MWT only 5 had reported the abovementioned type of respiratory involvement (8%).

## Discussion

The identification of the most appropriate and disease-specific outcome measures has become crucial for depicting disease natural history and assessing the efficacy of an experimental drug. In the last years, the number of available questionnaires or scales in the neuromuscular field has increased dramatically, so that the choice of which assessment to use is becoming challenging. The main measurement properties that need to be taken into account, to evaluate the performance and appropriateness of an outcome measure, are feasibility, content validity, internal consistency, criterion validity, construct validity, reproducibility, longitudinal validity, responsiveness, flooring and ceiling effects and interpretability (21). Some of these properties might greatly change depending on the target population to be tested. The aim of our study was therefore to test the applicability of the most commonly used motor outcome measures in the DM2 population. The main disease-specific domains that we intended to test were: myotonia, myalgia, muscle weakness, motor function, and endurance. Additionally, the balance domain was also investigated. For each domain, the assessment measures were selected based on our former systematic review of the literature on this topic (12). The presence of myotonia was assessed with the MBS including the hand opening time. The MBS scale was developed for patients with myotonia congenita, who mostly show a severer grip myotonia in comparison to DM2 (22). In a recently published study, MBS has been adopted also in DM1 and DM2 patients to validate the relaxation time on a dynamometer as measurement of myotonia (23). The results of our study were similar to those recently published by Horakova et al. as we found a mean MBS score of 1.5 (vs. Horakova 1.6). Interestingly, even if we haven't used a dynamometer to assess the hand opening time, we still found a similar mean value of 0.6 s in comparison to 0.4 s of that study. The good correlation between MBS and hand opening time point toward a good convergent validity and makes the MBS a suitable assessment method for myotonia in DM2. The high correlation between MBS and pain questionnaires, actually measuring a different domain, might indicate that some patients have difficulties in discriminating between myotonia, stiffness and pain, symptoms that often overlap. On the other hand, the INqoL questions on myotonia did not correlate with MBS and hand-opening time and some patients did not agreed on the term “muscle locking.” These questions however are part of a more comprehensive and fully validated questionnaire and their use detached from the whole INQoL has not been validated (24).

Being able to objectively measure myalgia represents a major challenge for the whole research field of chronic muscle pain. The most commonly used assessment tools are questionnaires that variably measure the intensity, quality and distribution of pain. Hand-held algometers, in their digital or analog version, are widely used to determine pressure pain threshold (PPT) either as sole assessment or in the context of the more complex and time-consuming quantitative sensory testing (QST). In our study we found the results of PPT to be extremely heterogeneous among repeated measurements. Furthermore, no correlations were found with the pain questionnaires and the mean PPT values could not discriminate DM2 patients with and without myalgia. PPT has been previously investigated in three studies on DM2 performed in comparison to healthy controls and/or fibromyalgia patients (25–27) with contradictory results. Several caveats related to the use of an algometer are responsible for these highly variable results: fat composition of the tested area, rate of application, experience of the examiner, size of the footplate and angle of application over the muscle (28). In the light of these considerations, the use of PPT in DM2 patients should be critically pondered. At the moment, deep knowledge of the pathophysiological mechanisms of pain in DM2 and its appropriate assessment still represent unmet needs. Peripheral mechanisms of pain possibly involving small nerve fibers might be postulated (25), even though the qualities of reported pain in DM2 patients in our and previous studies differ from the classic neuropathic pain of small fiber neuropathies. Further investigations with QST or skin biopsy in DM2 might add useful information.

The choice to test, for the first time, the utility of the SARA scale in DM2 was made after DiPaolo et al. validated it for DM1 (6). As expected, however, DM2 patients reached very low scores and very high flooring effect, up to 50.7%. The item responsible for about 40% of the test variance was “gait,” whereas other items as “speech,” “finger chase,” and “finger-nose” had very low eigenvalues and, if removed, would lead to an increased Chronbach's α. These results reflect well the known phenotypical differences between DM1 and DM2, as this latter group of patients usually does not have any prominent bulbar involvement, nor distal muscle weakness, which might greatly affect gait stability and upper limbs dexterity/movements in DM1 SARA scores.

The presence of muscle weakness is the most prevalent symptom in DM2 and increases with age, for this reason the proper assessment of muscle strength and motor function is crucial in monitoring disease progression. Two assessment methods of maximal isometric strength are adopted in neuromuscular diseases: the manual muscle testing (either grades 0–5 or modified by Kendall 0–10) and the hand-held dynamometry. Both assessments have been widely validated in many neuromuscular diseases and are considered complementary; in fact, the MMT is less time-consuming than HHD but also less sensitive and more operator-dependent (29). A direct comparison between the two methods has been mainly performed on healthy subjects and on few neuromuscular diseases retrieving contradictory results as regards concordance between MMT and HHD (30). We found a moderate to strong correlation for nearly all muscle groups investigated. Comparing the results of MMT and HHD at V0 and V1 a significant worsening was observed only with the HHD, thus confirming its higher sensitivity to change in comparison to MMT and making it a more suitable instrument for clinical trials or follow-up for such a slowly progressive disease. To evaluate the motor function, we have tested two scales that have been validated in late-onset Pompe disease: the GSGC and the QMFT (14, 15). Both scales showed a good convergent and differential validity in DM2, however only the QMFT showed acceptable percentage of ceiling effect (14.5%) (21). The higher ceiling effect observed by adopting the GSGC scale, may be explained by the lower number of items explored (4 vs. 16 of QMFT), investigating only the lower limbs and depicting higher levels of disability, more prevalent in Pompe disease than DM2. Both scales allowed the discrimination of different levels of disability, being able to distinguish patients with low disability (walking unaided) from patients needing a cane or a wheelchair. Therefore, the QMFT should be included in the development of a DM2 severity score, and as a useful tool to predict disability/need of walking aid.

The 6MWT is a well-established measurement of endurance, widely adopted to monitor patients with neuromuscular or cardiorespiratory diseases. It has been validated in many neuromuscular diseases (e.g., Pompe disease, FSHD, Duchenne and Becker dystrophy, spinal muscular atrophy, DM1) but not yet in myotonic dystrophy type 2 (7, 31–34). In our study the mean distance walked on 6MWT was higher than what reported in previous studies on DM1, this might be surprising considering the more proximal involvement of DM2 but is well-explained by the usually milder phenotype (8). The 6MWT showed an excellent convergent validity correlating with nearly all other motor outcome measures for lower limbs. No correlation was found as regards the presence of myotonia and myalgia, thus suggesting that these to symptoms do not greatly impact patients' performances on 6MWT. The worsening of the 6MWT of 34 meters at V1 is of difficult interpretation. In our study we could not perform solid test-retest analyses for 6MWT, thus no standard error of measurements (SEM) and minimally clinically important difference (MCID) could be calculated. In the literature, mainly regarding chronic cardiorespiratory diseases, the MCID ranges between 24 and 55 m, so that our value as those of other studies on Pompe disease and Duchenne dystrophy would fall within this range (31, 35).

Another measure of muscle endurance for lower limbs is the 30-s sit-to-stand test, especially used in the elderly population. This test allows easily assessing strength, function and endurance of lower limbs and it can predict the risk of falls in elderly (16, 36). The median number of repetitions performed by our patients was 9, which is lower in comparison to literature data for healthy older adults aged 60–70 (14 repetitions) and 70–80 (12 repetitions). The risk of falls increases in healthy elderly with scores <11 (36), our results would then match the recently reported higher risk of falls in DM1 and DM2 (37). Nevertheless, we have not systematically assessed the frequency of falls in our cohort reported here. For this test, it has also been recently developed an application, which uses inertial signals of a smartwatch to count the test repetitions; its use in myotonic dystrophy patients might be explored (38). About 20% of patients could not perform the test due to the severe weakness, thus hindering its application in more affected patients.

The Functional Index-2 was included in our test battery because it is a useful endurance test for upper limbs in myositis patients, being able to detect the presence of muscle impairment not caught by MMT (39). In this recent myositis study, the sole subcomponent of shoulder abduction has also been validated. In our study, a high percentage of patients (33%) reached the maximum score and the mean scores obtained were significantly higher than what reported for myositis patients suggesting that endurance at upper limb is not severely affected in the majority of DM2 patients.

This is to our knowledge the first prospective longitudinal study on motor function in DM2. Thus, far, only Sansone reported the results of MMT performed at baseline and after a mean of 7 years follow-up, but no regression analysis studies have been performed, so that the mean decline per year remains unknown (40). Comparing our results of V0 and V1, the HHD, the QMFT, and the 6MWT are the most sensitive to change, since all pointed toward a slow worsening of the clinical symptoms after 1 year. Longer follow-up visits are needed in order to assess the true muscular deterioration over time.

Among the limitations of this study, two main elements might have influenced the composition of our cohort. Firstly, a selection bias has probably occurred; it would be reasonable to think that patients with higher disability were not willing to travel to the study center and undergo the proposed test battery. A second element to be taken into account is the higher prevalence of female patients (62.9%) in our cohort, even though no significant differences were present as regards age and disease duration between males and females. Due to the 12-months time interval between V0 and V1, and the use of different evaluators, no reliability tests could be performed in this study and the validation of these tests mainly relies on feasibility, content validity, internal consistency, construct validity, flooring/ceiling effects and interpretability. The decline observed at V1 needs be further investigated with longer longitudinal data.

In conclusion, these preliminary data identify as most suitable motor outcome measures for DM2: the MBS for myotonia; the use of pain questionnaires for the description of myalgia; both the MMT and the HHD, according to the clinical setting (regular follow-up or clinical trials) for strength measurement; QMFT as global motor function assessment; 6MWT for the assessment of muscle endurance. Longer follow-up visits are needed, in order to assess the muscular deterioration over time. Further detailed data analyses of the patient reported outcomes will be reported in a separate manuscript.

## Data Availability Statement

The datasets generated for this study are available on reasonable request to the corresponding author.

## Ethics Statement

The studies involving human participants were reviewed and approved by Ludwig Maximilians University Ethic commission. The patients/participants provided their written informed consent to participate in this study.

## Author Contributions

FM contributed to the conception of the study, analysis and interpretation of the data, drafting the manuscript. ER contributed to conception of the study, data collection, analysis and interpretation of the data, review of the manuscript. KS and NK contributed to data collection and review of the manuscript. RM and BS contributed to conception of the study, interpretation of the data, and review of the manuscript.

## Conflict of Interest

The authors declare that the research was conducted in the absence of any commercial or financial relationships that could be construed as a potential conflict of interest.

## Abbreviations

10MWT: 10-meter walk test
30CST: 30-sec chair-stand test
6MWT: six-minutes walking test
BPI-sf: brief pain inventory short form
DM1: myotonic dystrophy type 1
DM2: myotonic dystrophy type 2
FI-2: functional index version 2
FDSS: fatigue and daytime sleepiness scale
GSGC: gait stairs Gowers chair
HHD: hand held dynamometry
INQoL: Individualized neuromuscular quality of life
MBS: myotonia behavior scale
MMT: manual muscle testing
NHPT: Nine Holes Peg Test
OMs: outcome measures
OMMYD: Outcome Measures in Myotonic Dystrophy type 1
PPT: pressure pain threshold
PROs: patients' reported outcomes
QMFT: quantitative motor function test
QMT: quantitative muscle testing
R-Pact: Rasch-built Pompe-specific activity scale
SARA: scale for the assessment and rating of ataxia.
